# The Role of ANRIL in Atherosclerosis

**DOI:** 10.1155/2022/8859677

**Published:** 2022-02-09

**Authors:** Iman Razeghian-Jahromi, Ali Karimi Akhormeh, Mohammad Javad Zibaeenezhad

**Affiliations:** Cardiovascular Research Center, Shiraz University of Medical Sciences, Shiraz, Iran

## Abstract

There is a huge number of noncoding RNA (ncRNA) transcripts in the cell with important roles in modulation of different mechanisms. ANRIL is a long ncRNA with 3.8 kb length that is transcribed in the opposite direction of the INK4/ARF locus in chromosome 9p21. It was shown that polymorphisms within this locus are associated with vascular disorders, notably coronary artery disease (CAD), which is considered as a risk factor for life-threatening events like myocardial infarction and stroke. ANRIL is subjected to a variety of splicing patterns producing multiple isoforms. Linear isoforms could be further transformed into circular ones by back-splicing. ANRIL regulates genes in atherogenic network in a positive or negative manner. This regulation is implemented both locally and remotely. While CAD is known as a proliferative disorder and cell proliferation plays a crucial role in the progression of atherosclerosis, the functions of ANRIL and CAD development are intertwined remarkably. This makes ANRIL a suitable target for diagnostic, prognostic, and even therapeutic aims. In this review, we tried to present a comprehensive appraisal on different aspects of ANRIL including its location, structure, isoforms, expression, and functions. In each step, the contribution of ANRIL to atherosclerosis is discussed.

## 1. Introduction

Coronary artery disease (CAD) has been become the leading cause of hospitalization and death in the era of cardiovascular disorders [1]. Among a variety of risk factors, genetic susceptibility is a determining one for initiation and progression of CAD [2]. Exploring the effects of differential gene expression helps to elucidate less known aspects of CAD pathogenesis, ultimately leads to efficient treatment and even, prevention [3]. One of the recently discovered gene-born elements with outstanding impact on gene expression is noncoding RNAs.

Only about 2% of the transcribed RNAs translate into proteins [4]. The remaining 98% are found as the parts of spliceosome, telomerase machinery, or ribosomal RNA. Furthermore, there are noncoding RNAs that are appeared in the form of short (microRNAs) or long noncoding transcripts (lncRNA) [4]. Usually, the length of lncRNAs is about more than 200 nucleotides [5]. Researchers thought that they are only a kind of transcriptional noise, but later, it was revealed that they are able to regulate different biological mechanisms. In fact, they are means of information transport between cells [6–8]. lncRNAs are much more complex than microRNAs regarding their functions like gene regulation, either positive or negative, altering chromatin structure, and contribution to developmental processes [9, 10]. Moreover, this type of RNAs implicates in several physiological and pathological processes such as cell growth and inflammation as well as regulation of cardiac development [5, 11–15].

Reasonably, lncRNAs could be useful diagnostic biomarkers and therapeutic targets since their expression is changed in inflammatory conditions such as CAD [16, 17]. Imbalance in the expression of a variety of lncRNAs and miRNAs is one of the hallmarks of atherosclerosis pathogenesis [18]. For instance, high expression of let-7 miRNA in the cardiovascular system could be assumed as an evidence for its important role in vascular-originated disorders. Moreover, the expression of let-7b is influenced by ANRIL verifying its role in the atherosclerosis [19]. ANRIL, an lncRNA, greatly involves in the expression of CAD-related genes playing a promising part in atherosclerosis development. Large unknown avenues in this area prompted us to present a comprehensive appraisal on different perspectives of ANRIL including its locus, exons, isoforms, expression, abundance, and functions in relation to atherosclerosis.

## 2. ANRIL and 9p21locus

Strong evidence showed that susceptibility to atherosclerotic vascular disease could beat least partly heritable. According to the findings from genome wide association studies, certain polymorphisms on chromosome 9p21 are associated with atherosclerotic vascular disease, CAD, stroke, myocardial infarction (MI), and aortic aneurysm, independent of well-known risk factors like hypertension, obesity, smoking, or dyslipidemia [20, 21]. Three genes encoding for tumor suppressor proteins are located in the INK4/ARF locus of the chromosome 9p21 [3]. If antiproliferative effect of these three suppressor molecules becomes downregulated, pathologic monocytosis or vascular proliferation is promoted facilitating development of vascular atherosclerosis [22, 23]. To substantiate, p16-knock out mice demonstrated increased vascular hyperplasia after intra-arterial injury [24]. Also, it was seen that formation of atherosclerotic plaque is augmented in the case of ARF deficiency [25]. In contrast, induction of p16 and p15 levels possesses antiatherogenic effects [26–28]. All the encoded proteins in this region play critical roles in the regulation of cell proliferation, which is a determining phenomenon in the pathology of atherosclerosis [29].

**Figure 1:**
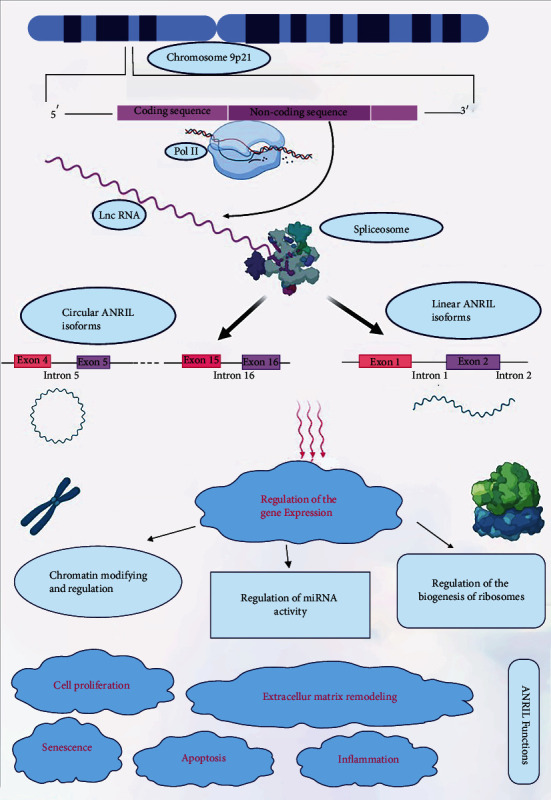
(i) ANRIL is located at the human CDKN2A/B locus at 9p21 which is transcribed by RNA polymerase II and spliced into multiple linear and circular isoforms in a tissue-specific manner. (ii) ANRIL has important well-established roles in cell proliferation, apoptosis, senescence, inflammation, and extracellular matrix remodeling through regulation of the gene expression via regulation of the miRNA activity, the biogenesis of the ribosomes, and chromatin modifying.

ANRIL (Antisense Noncoding RNA in the INK4 Locus), also known as CDKN2B-AS or CDKN2B-AS1, is a 3.8 kb lncRNA that is transcribed in the antisense direction of this gene cluster [30, 31]. Recently, ANRIL has been the point of great attention because it has been characterized as the main element in the chromosome 9p21 CAD locus [32, 33]. Generation of ANRIL and its diverse biological functions are schematically depicted in Figure 1. Most of the studies reported a link between ANRIL expression and Chr9p21 genotype [34]. In fact, nucleotide polymorphisms (SNPs) of this region significantly contribute to the different expression levels of ANRIL [35]. Genetic variants associated with atherosclerosis lie within the ANRIL codons [36]. This diversified expression imposes different effects on cis- and trans-gene regulation [19]. Certain polymorphisms of ANRIL like rs4977574, rs1333040, rs1333042, and rs10757274 are associated with CAD or increasing the risk of MI. ANRIL rs4977574 A > G is possibly a strong risk factor toward developing MI or CAD. It was revealed that rs1333040 T allele is an indicator of genetic susceptibility for CAD especially in the Asians [37]. Some ANRIL polymorphisms like rs1004638, rs1333048, and rs1333050 may be the genetic biomarkers of CAD but not MI or acute coronary syndrome [38]. DNA methylation and binding of transcription factors at the ANRIL promotor region are affected by the existing alleles, which in turn affects the expression level of ANRIL. This, on the other hand, demonstrates another level of regulating mechanisms for the expression of ANRIL [38]. Interestingly, other than SNPs within this locus, expression of the INK4/ARF products is influenced by polymorphisms located at about 120 kb away [36].

Polycomb group complexes control the INK4/ARF locus via inducing repression [39]. This type of control is important for maintenance and proliferation of somatic stem cells and self-renewal of tissues [40, 41]. SNPs at this locus alter the repression intensity which may increase the individuals' vulnerability to atherosclerosis following the changes in ANRIL expression or its splicing pattern [36]. Different splicing patterns cause certain modifications in ANRIL structure which in turn modifies the repression intensity by Polycomb group complexes [36]. There is strong correlation between these three: (1) expression of different isoforms of ANRIL proximal to the INK4/ARF locus, (2) INK4/ARF transcription, and (3) the risk of atherosclerotic disease. However, distal variants of ANRIL involving exons 18 and 19 are independently expressed with no correlation with INK4/ARF transcripts [36].

## 3. ANRIL and Exons

Earlier, it was assumed that ANRIL has 19 exons [42]. With the new discoveries in 2017, the number of exons increased to 21 [36]. In healthy subjects, lymphocytes and monocytes both express ANRIL [19]. Four major groups of ANRIL with common proximal exons (exons 1, 5, and 6) and various lengths were found in human peripheral blood mononuclear cells (8 as 9). Most of the exons have less than 100 nucleotides long. Many of them entirely consist of repetitive long- and short interspersed nuclear elements (LINE and SINE) as well as *Alu* elements [43]. Existence of several LINE and SINE elements besides repetitive motifs modulates ANRIL splicing [44, 45]. Splicing is also influenced by ANRIL localization. For example, those expressed in the vascular smooth muscle cells (VSMCs) contain the last exons and lack the first ones leading to a different splicing pattern [46].

RNA polymerase II transcribes ANRIL, which is then spliced into numerous linear and circular isoforms [32]. Exons in the linear and circular isoforms are somewhat different. While proximal exons [1, 2] are mainly found in linear isoforms, circular ones are more likely to contain central exons [4–16, 36]. Downregulation of the first exons is associated with some alleles that are related to vascular diseases. For instance, CCA phenotype is related to reduce ANRIL expression [46]. The majority of SNPs in the 9p21 locus were detected in the region of 13-19 exons in ANRIL [47, 48]. All exons at the INK4/ARF locus are expressed at very low levels, several folds lower than their protein peers (p15 and p16) [36].

## 4. ANRIL Isoforms: Linear and Circular

Complex tissue-specific splicing of ANRIL results in the production of multiple isoforms [32, 36, 49]. Different cell types may have different isoforms, circular or linear. In any cell type, several isoforms have been identified albeit at low amount [32, 50, 51]. For instance, two, three, and five isoforms are expressed in testes, lung, and human umbilical vein endothelial cells (HUVECs), respectively [42, 49]. No isoforms of ANRIL are predominant *in vivo* [36]. All the linear or circular ANRIL isoforms contribute positively or negatively to the atherosclerosis with different degrees (18-20 as 10). Footprints of ANRIL are found in different atherosclerosis components including vascular endothelial cells, VSMCs, mononuclear phagocytes, and atherosclerotic plaques [29, 52–54]. In various cell types, many typical linear polyadenylated ANRIL isoforms have been discovered [32]. At least 20 linear isoforms of ANRIL have been identified at Chr9p21 [50, 51, 55]. The expression level of diverse transcripts of linear ANRIL has various impacts on gene regulation [35]. Linear transcripts have been linked to an increased risk of atherosclerosis [35]. The proximal linear CDKN2B-AS1 transcripts have been demonstrated to play a role in the development of diabetes and CAD [56]. Upregulation of *in vitro* linear ANRIL expression increases proatherogenic cell activities like proliferation and reduced apoptosis, as well as differential expression of hundreds of genes, without impacting the expression of CDKN2A/B suppressors [34]. Indeed, the severity of atherosclerosis was positively linked with linear ANRIL levels [46, 47, 57].

### 4.1. Circular ANRIL

Circular RNAs are noncoding ones with covalently closed ends. Although discovered in the 80th decade, most of the researches on the circular RNAs have performed in the recent years. They are more stable than the linear counterparts, present in all the eukaryotes, and their expression is tissue-specific. Circular RNAs implicate in apoptosis, oxidative stress, defense mechanisms against microbial infection, and totally maintaining cellular homeostasis. While some of the circular RNAs behave like sponge or decoys to sequester RNA/protein complexes, others may provide scaffolds for the formation of protein complexes. Most of the circular RNAs are low in amount in relative to their targets [58, 59]. For instance, only 800-1000 copies of the circular ANRIL per cell inhibit rRNA processing factor, which is present at about 10 folds higher [50, 60].

Chromatin regulation is also performed by circular RNAs [61]. The function of the circular RNAs in promoting or preventing cellular stress is possibly carried out through inhibition of miRNA activity via induction of homeostasis or enhancing stress response as an adaptation mechanisms to chronic conditions [60, 62]. In particular, ANRIL and miRNA networks have collaborations with each other in gene expression as well. ANRIL regulates miRNA transcription epigenetically and also binds to the miRNA as a sponge. ANRIL expression is negatively related with its target miRNAs in the tissues and cell lines [63–68]. Sponging miRNAs give ANRIL prooncogenic effects. However, some ANRIL isoforms like those with exons 5-6-7 lack sponge activity [50]. It should be noted that most circular RNAs are present in the cytoplasm [69, 70]. Circular variants could be quantified by PCR (with outward-facing primers and specific exon-exon junction approach), or based on their tolerance toRNAse R digestion [32].

Circular form of ANRIL is produced upon back-splicing of linear transcripts. Back-splicing is the joining of a downstream splice donor site to an upstream splice acceptor site [71, 72]. It seems that generation of circular ANRIL is a competitive process against linear peers [73]. During stress conditions, splicing pattern changes from sequential to back-splicing in order to produce circular forms. So, all the circular forms may have no specific functions, and they are just a byproduct of alternative splicing. It was shown that linear RNAs are expressed at 10 folds lower than their circular counterparts [74, 75]. Binding of circular RNAs to the transcription factors regulates RNA splicing [76]. Circular RNAs themselves may undergo another round of splicing which is a unique characteristic of the circular isoforms [77, 78]. However, circular and linear ANRIL have common properties like enhanced stability and longevity [79].

**Figure 2:**
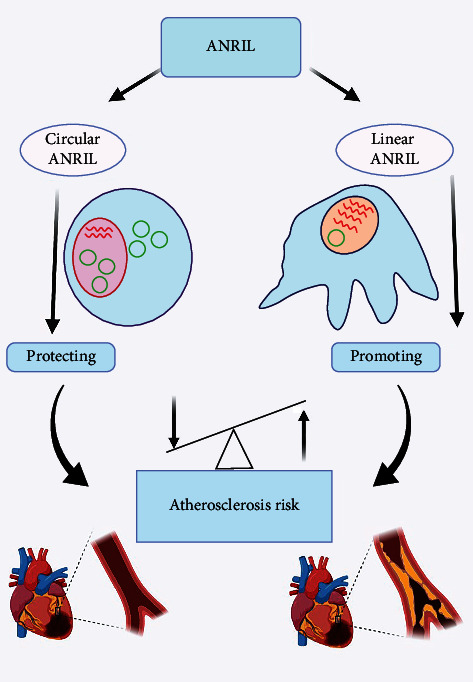
The ANRIL isoforms are associated with cardiovascular diseases. Linear ANRIL isoforms are related to increase risk of atherosclerotic plaques and promotion of atherosclerosis risk. On the contrary, circular ANRIL isoforms contribute protection of atherosclerotic plaques and atherosclerosis risk.

Novel circular ANRIL isoforms have been identified whose expression levels are in association with transcription level of the INK4/ARF locus, and hence, they are in close relation with atherosclerosis risk [36]. Expression of circular ANRIL is altered due to SNPs in the 9p21 locus as well. High circular and low linear activity lead to atheroprotection. The proportion of expression between circular and linear ANRIL determines development of atherosclerotic plaques. It means that cell uses circularization as a way to avoid synthesis of linear forms preventing plaque growth (Figure 2) [47, 80, 81]. Circular ANRIL regulates miRNA expression in the atherosclerotic plaques which might affect its growth [18]. Coronary atherosclerosis is prevented by reducing the expression of circular ANRIL via decreasing apoptosis in vascular endothelial cells and attenuating the expression of inflammatory factors [82]. Downregulating the expression of circular ANRIL is a new phenomenon in diagnosis and treatment of CAD because inhibition of this molecule reduces vascular endothelial injury, oxidative stress, and inflammatory responses [83].

Since circular ANRIL shows heterogenous expression in primary smooth muscle cells and macrophages of the vascular tissues, scrutinizing ANRIL functions in single cell level provides insightful points toward elucidation of atherosclerosis pathogenesis [50].

While circular RNAs have no capacity to produce proteins, they have no association with ribosomes [72, 84]. Nonetheless, they regulate biogenesis of ribosomes in VSMCs [50]. Also, they modulate apoptosis and proliferation of vascular cells conferring atherosclerosis protection [50]. This type of ANRIL prevents rRNA maturation via binding to lysine-rich domain of pescadillo zebrafish homologue 1 (PES1) [85]. Consequent impairment in the biogenesis of ribosome activates p53 leading to increased apoptosis and decreased proliferation. Protein translation and cell growth are decreased, totally result in reducing the number of proliferating cells in the atherosclerotic plaques yielding atheroprotection [6].

. However, a circular ANRIL isoform was identified that increases the risk of atherosclerosis. This isoform contains exons 5, 6, and 7 involving in rRNA maturation and nucleolar stress induction [50]. Circular ANRIL has many prominent characteristics including stability against degradation and features of antiatherogenicity and antiproliferativity. These properties make this form of ANRIL a valuable candidate for therapeutic approaches in proliferative disorders like atherosclerosis [50]. Moreover, being stable for several days gives a buffer-like face which is useful in stress conditions [86].

## 5. ANRIL Expression and Abundance

ANRIL downregulation showed positive effects in animal models of diabetes in terms of decreasing body weight, blood glucose level, and islet cell apoptosis [87]. Expression of ANRIL in different types of tumors shows its prominent role in cellular proliferation and apoptosis which are important steps of atherosclerosis [19]. Impaired expression of ANRIL in the vascular endothelial cells is associated with inflammation which is followed by acceleration of endothelial injury [33]. Also, proliferation, migration, senescence, and apoptosis of VSMCs are influenced by abnormal expression of ANRIL [88].

Activity of transcriptional promoter, alternative splicing, and RNA stability determine ANRIL abundance. Epigenetic regulation via methylation of promoter region imposes long-lasting effects on ANRIL-related gene expression and subsequent tissue functions [89–91]. Specific transcription of ANRIL in each cell type regulates specific sequels in the cellular processes. Another level of regulation is implemented through splicing that leads to multiple varieties and dissimilar ANRIL abundance in different cell types [32]. Furthermore, some elements like age, diabetes, and hypertension, traditional CAD risk factors, affect ANRIL level [92]. However, it was shown that lipid profile has no association with ANRIL expression [93]. Some cellular processes like genotoxic stress, tumorigenesis, senescence, and inflammation also influence ANRIL expression [32].

## 6. ANRIL Functions

Intriguingly, there is no homolog for ANRIL transcript in mice. Seemingly, ANRIL functions are specific to the humans [46]. The 86 codons of the longest open reading frame in ANRIL isoforms reinforce this fact that ANRIL functions are delivered through RNA activity [49]. The main function of ANRIL is regulation of gene expression [3]. Cell proliferation, senescence, apoptosis, extracellular matrix remodeling, and inflammation are all influenced by ANRIL activity [94].

It was reported that linear ANRIL containing proximal (exon 1) and distal (exons 13b and 19) exons were the predominant isoforms in the nucleus of melanoma cells. Both linear and circular ANRILs with middle exons [5–7] are present in the cytoplasm [51]. Nuclear localization of linear variants is an evidence for certain functions such as regulation of gene transcription via chromatin modulation while homing of circular isoforms in the cytoplasm shows posttranscriptional functions [32]. It was reported that overexpression of an ANRIL variant activates some genes that involve in the architecture of the nucleus and chromatin [95].

Inflammation and its components like chemokines, cytokines, and growth factors substantially involve in CAD pathogenesis [96]. Linkage of CAD to inflammation is a fact that has been confirmed in several studies [97–99]. A regulatory role is considered for ANRIL which bridges CAD and inflammation [33]. Proinflammatory factors like NF-k*β* and TNF-*α* upregulate ANRIL in endothelial cells showing its relation to inflammation [32]. In turn, ANRIL upregulation modulates the expression of inflammatory gene downstream of NF-*κ*B. ANRIL binds to a transcriptional component (Yin Yang 1) to form a functional complex exerting transcriptional regulation on inflammatory genes like IL6 and IL8 [33]. In fact, ANRIL is known as a novel member of TNF-*α*-NF-k*β* pathway that activate inflammatory elements in the pathological conditions [33].

As antisense of CDKN2B, ANRIL has an inhibitory effect on the expression of sense sequence [46]. ANRIL recruits polycomb repressive complexes to their promoter regulating the expression of protein-coding genes [68, 100]. There is an inverse relationship between ANRIL and CDKN2B expression. Downregulation of ANRIL is concomitant with the upregulation of CDKN2B which has an antiproliferative effect resulting in reduced proliferation [46]. In contrast, expression of ANRIL, CDKN2A, and CDKN2B is reported to be in a positive correlation with each other in other investigations and shows that transcription of these genes is coregulated in many tissues [36, 43, 49, 94, 101–104]. During cell growth, polycomb proteins repress CDKN2A/B region via histone modification while they are activated in senescence [100, 105, 106]. That is why the expression of CDKN2A is increased with age [107]. Reasonably, ANRIL is closely associated with cell senescence as well [106]. An interesting finding is that ANRIL is in a stronger association with phenotype in some cases compared with CDKN2A/B protein-coding genes [102, 108].

Acting as a scaffold, ANRIL helps to form complexes that regulate target genes via histone modification, ROS upregulation, and aortic phenotype transition. This novel epigenetic regulation provides extra insights for ANRIL to be a therapeutic target in occlusive vascular diseases [37]. Phenotypic change of VSMCs is a determinant factor in atherosclerosis development. Regulation of the functions of endothelial cells and VSMCs by ANRIL [109, 110] remarkably contributes to vascular homeostasis [111]. In fact, ANRIL alters the activity of AMP-activated protein kinase (AMPK) which subsequently prevents phenotypic switching of VSMCs inhibiting plaque formation [111]. Ox-LDL induces the expression of ANRIL as well as ROS promoting phenotypic transition of human aortic smooth muscle cells [112]. In this regard, both ANRIL and AMPK could be considered as therapeutic targets for attenuating atherosclerosis-associated vascular diseases [111].

ANRIL facilitates atherosclerosis progression via sponging mir-399-5p and regulating RAS/RAF/ERK signaling pathway. This finding demonstrates the impact of ANRIL/mir-339-5p/FRS2 regulatory axis on the oxLDL-induced human aortic VSMCs and HUVECs progression [113]. It was shown that upregulation of ANRIL results in induction of proliferation and inhibition of apoptosis in human coronary endothelial cells and HUVECs in a mir-181b-dependent manner [114]. Cytokines of IL-10 and MCP-1, which are known as the markers of endothelial dysfunction, are associated with ANRIL expression [19]. Regulation of endothelial dysfunction by ANRIL is performed through inhibition of HUVEC proliferation and angiogenesis, promotion of apoptosis, and activation of inflammation which all are mediated by controlling the let-7b/TGF-*β*R1 signaling pathway [19].

Regarding the association between ANRIL and plaque stability, downregulation of ANRIL expression attenuates endothelial cell dysfunction, augments the proliferative and angiogenic capacity of damaged endothelial cells, promotes the expression of anti-inflammatory mediators, decreases local inflammation of the plaque, and increases the stability of the plaque fibrous cap [19]. In particular, ANRIL is important for regulation of two tumor suppressor genes, cyclin-dependent kinase inhibitors A and B in CDKN2A/B locus that are involved in atherosclerosis in terms of thrombogenesis, vascular remodeling or repair, and plaque stability [32, 42]. High ANRIL expression was considered as an independent risk factor for in-stent restenosis. However, it was declared that the added diagnostic potential of ANRIL will be reached when combined with expression level of other lncRNAs like homeobox A11 antisense [115].

## 7. Concluding Remarks

The importance of the functions of lncRNAs in the normal cells becomes apparent when different disorders are emerged upon deregulation of such elements [116]. Close relationship has been found between the CDKN2A/B locus and a variety of well-known disorders such as atherosclerotic disease, type 2 diabetes, stroke, periodontitis, aging, and hypertension [101, 117]. Many disease-associated SNPs within or adjacent to the ANRIL gene have been identified [118]. It was even declared that ANRIL regulates its SNPs [3]. Abnormal expression of ANRIL facilitates the incidence of a range of atherosclerosis-related impairments including vascular endothelial injury, deteriorations in VSMCs, mononuclear cell adhesion/proliferation imbalance, glycolipid metabolism disorder, DNA damage, and competing endogenous RNAs [3].

All in all, the role of ANRIL in atherosclerosis results from a balance between the level of linear (atherogenic) and circular (antiatherogenic) variants [35]. Also, pathological changes raised by CAD affect ANRIL expression [34]. However, the main point is that molecular differences in ANRIL (linear vs. circular) may lead to a fundamental change in its function in terms of proliferation rate in smooth muscle cells and macrophages [34] in a way that a slight dominance of linear over circular ANRIL directs the path in favor of CAD [50].

The exact molecular orchestrate forced by the genotype variations in the relevant locus in which determines the ratio of linear over circular ANRIL is still not fully known. This may be due to the fact that both linear and circular isoforms always coexist with each other in the cell, and additionally, share the same sequences in part [34]. Since relative abundance of linear/circular ANRIL may be a determining factor for the atherosclerosis development [55], measuring this ratio may have predictive value, aids in CAD risk classification, and improves monitoring of treatment response as well as disease relapse. Also, it is still being studied if inhibiting linear ANRIL or increasing circularization is enough to protect against atherosclerosis *in vivo* [34].
